# A Co-Opetitive Automated Negotiation Model for Vertical Allied Enterprises Teams and Stakeholders

**DOI:** 10.3390/e20040286

**Published:** 2018-04-14

**Authors:** Taiguang Gao, Qing Wang, Min Huang, Xingwei Wang, Yu Zhang

**Affiliations:** 1 College of Information Science and Engineering, Northeastern University, Shenyang 110819, China; 2 State Key Laboratory of Synthetical Automation for Process Industries, Northeastern University, Shenyang 110819, China; 3 School of Management, Heilongjiang University of Science and Technology, Harbin 150022, China

**Keywords:** vertical allied enterprises team, automated negotiation, non-cooperative game, Shapley value, supply chain, co-opetition

## Abstract

Upstream and downstream of supply chain enterprises often form a tactic vertical alliance to enhance their operational efficiency and maintain their competitive edges in the market. Hence, it is critical for an alliance to collaborate over their internal resources and resolve the profit conflicts among members, so that the functionality required by stakeholders can be fulfilled. As an effective solution, automated negotiation for the vertical allied enterprises team and stakeholder will sufficiently make use of emerging team advantages and significantly reduce the profit conflicts in teams with grouping decisions rather than unilateral decisions by some leader. In this paper, an automated negotiation model is designed to describe both the collaborative game process among the team members and the competitive negotiation process between the allied team and the stakeholder. Considering the co-competitiveness of the vertical allied team, the designed model helps the team members making decision for their own sake, and the team counter-offers for the ongoing negotiation are generated with non-cooperative game process, where the profit derived from negotiation result is distributed with Shapley value method according to contribution or importance contributed by each team member. Finally, a case study is given to testify the effectiveness of the designed model.

## 1. Introduction

When China entered a new stage of economic development after the financial crises in the years 2007–2008 and 2008–2012, namely the new normal economy, its economic growth shifted from high-speed to medium-to-high-speed, the economic structure was constantly improved and upgraded, and the economic development shifted from input-driven and investment-driven to innovation driven with the removal of red tape and delegation of powers to authorities at lower levels [1]. The new normal economy has led the supply chain management to be more agile and competitive in the global context, and it is no longer limited to the competition among single enterprises in market, but the competition between supply chains has become the mainstream trend of the current economic development [2,3]. To maintain their own competitive edge, improve the utilization of idle resource, and reduce duplicate investment and operational risk, enterprises often undertake vertical alliances with upstream and downstream ones in the supply chain as a business tactic in their competitive strategy [4,5,6]. Hence, the allocation of idle resources and the formation of scale economies in the supply chain can be improved, which also is advantageous to enhance the coordinated development level of the supply chain [7]. As a result, it is critical for an alliance to collaborate over internal resources and resolve the profit conflicts among members, so that the functionality required by stakeholders can be fulfilled. As an effective means to resolve the profit conflicts and realize the coordinated development of supply chain, negotiation has received a great deal attention of academic and practitioners such as the collaborative planning among supply chains partners [8] and eco-efficient or open innovation in a supply chain [9,10,11,12,13,14] etc. However, the negotiations in industries are often inefficient due to the diversity of intellectual backgrounds of the negotiating parties, many variables involved, complex interactions, and inadequate negotiation knowledge of the project participants [9,14,15]. In recent years, with the rapid development of Internet and information technology, automated negotiation based on multiple agents is widely studied and applied to the operations and management of supply chain, which provides an effective way to resolve various conflicts in it [4,5,6]. Considering the challenge for the users in terms of quality-oriented selection of their required services, Hashmi et al. [16] presented a social network-based trust framework (*SNRNeg*) and proposed an end-to-end automated negotiation approach of Genetic Algorithm-based Web service for modeling dependency relationships among the Quality of Service (QoS) component of Web services with static environment. Patrikar et al. [17] proposed a linear programming and pattern matching based multilateral automated negotiation system and studied some multilateral system with several methods. To find a proficient mechanism helping an agent to decide under which conditions to accept opponent’s offer in a bilateral automated negotiation, Baarslag et al. [18] compared the performance of various acceptance conditions in combination with a broad range of bidding strategies and negotiation scenarios, and then proposed new acceptance conditions meanwhile demonstrated their advantages relative to other conditions by numerical experiments. To enhance the ability of an agent to quickly and autonomously select an appropriate strategy among the candidates according to the situation changes of automated negotiation, Cao et al. [19] developed multi-strategy selection theoretical model and algorithm, by the experimental results analysis, which may enable a negotiating agent to select appropriate strategy dynamically to deal with the ever-changing opponent’s offer and achieve a more efficient and effective agreement than possible with a conventional fixed strategy. In order to effectively coordinate the demand, production and business contracts or orders, Hernández et al. [20] proposed an automated negotiation mechanism suitable for multi-level supply chain coordination. However, with the assumption that each one is fully cooperative, their work is only limited to the study of the cooperation and negotiation problems in a supply chain, but the negotiation between the supply chain and demanders is not taken into account. Since big data may provide novel insights into, inter alia, market trends, customer buying patterns, and maintenance cycles, as well as into ways of lowering costs and enabling more targeted business decisions [21], firms can better understand customers’ preferences and needs by analyzing the big data generated from various sources, such as market information and the records of historic cooperation and negotiation etc., and managers increasingly view data as an important driver of innovation and a significant source of value creation and competitive advantage [22]. According to the historical information of a supply chain and market, Giannakis et al. [23] studied how to improve the agility of the supply chain by combining big data method with multi-agent automated negotiation, and designed a framework model, which may be impractical as assumed that all supply chain members are fully cooperative and they may share all obtainable information and resources each other [24]. In the research and application of big data, it is always the main challenges to deal with massive amounts of data and to leverage or apply predictive analytics, as it is extremely hard for existing analytic methods to process high volume of various data in real time and produce useful information [22,25], and it is also difficult to accumulate so much and efficient negotiation information or records with the same partner or the relevant operational data of supply chain management considering the trade secret and trust in practice. Thus, it is tough to resolve all decision-making problem by big data method which should be used as a supplementary approach for the research and application of automated negotiation and be suitable to underscore and shed some light on certain partial aspects. In recent decades, most of current works have focused on the processes of which parties (bilateral, or multiparty) are of single individuals, but some real-world scenarios may involve negotiation parties of more than a single individual [26,27,28]. The research carried out by Sheu and Gao [29] found that supply chain or allied members participate in a negotiation as a team can significantly improve their bargaining power in the negotiation, with regarding to cost reduction of operations, trading and technology innovation [12]. According to that, Sanchez-Anguix et al. [26,27,28] defined the supply chain members in negotiation as an allied team and proposed an automated negotiation model called Team Negotiation based on Profit Order Difference (*TNPOD*), in which each team member has an independent decision for the calculation of counter-offer, and the final counter-offer of the team was selected by members vote. However, the measurement and distribution of alliance collaboration profit cannot be considered in their work, and it is short of analyzing the game process in decision-making of the team. *The alliance collaboration profit* is the profit saved from the operational cost of supply chain by the effective collaboration and communication of members in the alliance. Inside the allied team, the game process of the decision-making on supply chain operations and management gradually becomes the focus of academic and applied research [3,30,31], but the special game problem with co-opetition is often ignored. Although, in reality, allied team members do expect that their profits can be improved by the cooperation in an alliance, it is difficult for them to completely share resources and operational information due to the fact that they are usually independent and self-financing economic entities [32,33,34,35,36], and each party must be aware of which information should be shared or not, because there is an objective difficulty in identifying trustworthy potential partners [14]. Thus, it is very necessary to study the special game problem in the supply chain alliance with co-opetition partnership.

This paper designs an Automated Negotiation model for the vertical allied Team (*ANT*), with a competitive negotiation process between the team and the stakeholder (a *stakeholder* may be any possible party or organization in a supply chain which might negotiate with the vertical allied enterprises team), and with a collaborative game process among the team members. The counter-offer process in the allied team is calculated from the perspective of a non-cooperative game, and the distribution of the alliance collaboration profit is of Shapley value solution in terms of the offer contribution rates. Finally, a case study is given to testify the effectiveness of the designed model, where the stakeholder to replenish coal products and the vertical allied team including one coal enterprise and one logistic enterprise are involved in the automated negotiation to make a business agreement. The major contributions of this paper are as follows: on the one hand, the decision-making of the vertical allied team with co-opetitive relationship is shaped with game process in the negotiation with opponent. On the other hand, with the designed automated negotiation model, the vertical allied team may properly manage internal collaborative business even competition coexists among team members. Furthermore, this model can help many allied teams in supply chain or independent organizations to make agreement with other stakeholders or potential cooperators.

The rest of the paper is structured as follows: Section 2 establishes a platform framework of automated negotiation with team-side game, and with regards to negotiation scenario two agent types are defined for the allied team and the individual members, respectively. In Section 3 a non-cooperative game process is designed for team members’ collaboration with Shapley Value solution. Section 4 demonstrates and testifies the effectiveness of the proposed model with the analysis of numerical experiments. Section 5 presents summary of this paper and the suggestions for the future studies.

## 2. Framework of the Automated Negotiation Model

### 2.1. Negotiation Scenario

#### 2.1.1. Problem Analysis

In reality, the cost of some products or services is usually dependent on the collaboration of production and logistics, and the logistic costs often account for a higher percentage of the total cost, such as in the case of fresh or perishable products, energy products including coal and oil and so on. Meanwhile the different quantity and completion times have a significant impact on the total cost of those products. Therefore, in order to improve the operational efficiency of supply chain and reduce additional costs and losses caused by undesired collaboration, the production and logistics enterprises of those products or services prefer to form a supply chain vertical allied team. Although all the allied team members are seeking to consolidate their market competitive advantages through the collaboration with upstream and downstream enterprises of supply chain, it is impossible for them to completely share their resources and operational information since each of them is independent and self-financing [24,37,38]. Hence, either non-cooperative or cooperative game processes for the team or its members cannot comprehensively reflect the characteristics of the vertical alliance with co-opetition partnership [30,35,36]. In this paper, without loss of generality, *the vertical allied team* or team for short is defined as a group of one *Production enterprise* and one *Logistics enterprise* in a supply chain, and the *Stakeholder enterprise* is defined as an independent entity, which seeks for the provider of some material, product or service such as a manufacturer, retailer or customer, etc. All team members are well familiar with each other’s productivity or strength, and many settings are in common sense such as *the pricing rules, collaboration profit function and the historical information* about negotiation. In this study, *three issues of quantity, price and delivery time are taken account in the model*. The value ranges of each issue thought by the team might be different from those of *Stakeholder enterprise*.

#### 2.1.2. Automated Negotiation Process of the Proposed Model 

The automated negotiation process is designed basing on any supply chain Business to Business (B2B) electronic platform. *Stakeholder enterprises* which have demands for fulfillment, *Production enterprises* and *Logistics enterprises* can enroll in the platform to seek business. To clearly describe the automated negotiation process, suppose that the B2B platform receives the request of a *Stakeholder enterprise* to meet a demand, it chooses a pair of *Production enterprise* and *Logistics enterprise* as a vertical allied team to carry out the demand.

**Figure 1:**
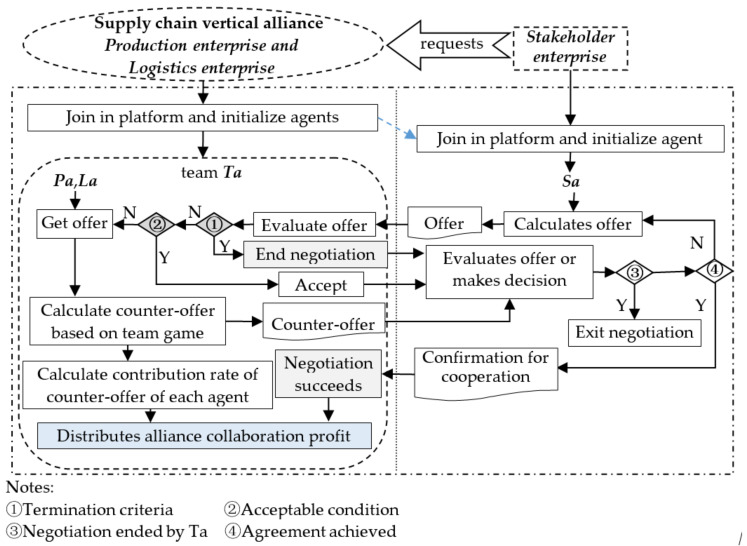
The process of automated negotiation.

The automated negotiation is arranged by B2B platform system for the *Stakeholder enterprise* and the team, and the agents for *Stakeholder enterprise*, the team with its *Production enterprise* and *Logistics enterprise* members are initialized as well, denoted by *Sa*, *Ta*, *Pa*, *La*, where *Ta* can also be seen as a trusted mediator or organizer agent of the team. In each round or iteration of automated negotiation between *Sa* and *Ta*, firstly, *Sa* sends an offer to *Ta*, then a counter-offer is generated by *Ta* basing on the outcome of the team-side game if the *Sa* offer is negotiable, repeating in this way until the negotiation succeeds or stop with termination criteria. With respect to each counter-offer the *Ta* distributes the deduced collaboration profit according to the contribution rates of *Pa* and *La* on the total profit over the counter-offer with Shapley value method. The process of automated negotiation is illustrated in Figure 1. It is worth to note that the automated negotiation will continue unless *Ta* refuses the *Sa* offer or agreement is achieved before the designated deadline.

### 2.2. Automated Negotiation Model ANT

Firstly, symbols and denominations are defined as in Table 1:

According to the characteristic of the negotiation between the allied team *Ta* and *Sa*, *ANT* can be defined as a 3-tuple:

(1) ANT=(I,Ta,Sa)

where *I =* {*q, p, d*} denotes the issues set concerned in the automated negotiation process. For the instance of above scenario, *q*, *p* and *d* denote the quantity, price and delivery time, respectively.

#### 2.2.1. Allied Team for Negotiation

According to the characteristic of the allied team in *ANT*, *Ta* can be defined as a 12-tuple:

(2) $$Ta=\left(A,T_{Ta},{\bar{q}}_{Ta},{\underline{q}}_{Ta},{\bar{d}}_{Ta},{\underline{d}}_{Ta},b_{Ta},p_{Ta},C_{Ta},\alpha_{Ta},\beta_{Ta},\phi_{Ta}\right)$$

where *A* = {*Pa, La*} is a set of member agents in the team; round-dependent price function *p_Ta_* can be represented as follows [39]:

(3) $$p_{Ta}^{t}(q_{Sa}^{t},\;d_{Pa}^{t},\;d_{La}^{t})=b_{Ta}\times \left(1-\frac{1}{2}\Delta_{Ta}^{t}\eta_{Ta}^{t}\right),0<q_{Sa}^{t}\leq {\bar{q}}_{Ta},{\underline{d}}_{Ta}^{}\leq d_{Pa}^{t},d_{La}^{t}\leq {\bar{d}}_{Ta},\forall t=1,\ldots ,T_{Ta}$$

where $q_{Sa}^{t}$ denotes the quantity requested by *Sa* at the *t-*th round; $\Delta_{Ta}^{t}=0$ if $0<q_{Sa}^{t}<{\underline{q}}_{Ta}$, $\Delta_{Ta}^{t}=\frac{q_{Sa}^{t}-{\underline{q}}_{Ta}}{{\bar{q}}_{Ta}-{\underline{q}}_{Ta}}$ if ${\underline{q}}_{Ta}\leq q_{Sa}^{t}\leq {\bar{q}}_{Ta}$; $n_{Ta}^{t}=\underset{i\in \left\{Pa,La\right\}}{\prod }\frac{d_{i}^{t}-{\underline{d}}_{Ta}}{{\bar{d}}_{Ta}-{\underline{d}}_{Ta}}$.

*C_Ta_* in (2) denotes the unit cost function:

(4) $$C_{Ta}^{t}(D_{Ta}^{t},q_{Sa}^{t},C_{Pa}^{t},C_{La}^{t})=\sum_{i\in \{Pa,La\}}C_{i}^{t}-\rho_{Ta}^{t},\forall t=1,\ldots ,T_{Ta}$$

where $\rho_{Ta}^{t}$ can also be interpreted as the profit obtained by the collaboration of member agents in *Ta* [36] if the quantity is $q_{Sa}^{t}$, which can be calculated by:

(5) $$\rho_{Ta}^{t}(q_{Sa}^{t})=\frac{1}{2}{\left(\Delta_{Ta}^{t}\right)}^{2}\times \sum_{i\in \{Pa,La\}}c_{i},\forall t=1,\ldots ,T_{Ta}$$

As a result, the total profit of the allied team is

(6) $$u_{Ta}^{t}=q_{Sa}^{t}\times \left(p_{Ta}^{t}-C_{Ta}^{t}\right),\forall t=1,\ldots ,T_{Ta}$$

When receives the *Sa* offer at the *t-*th round, $o_{Sa}^{t}=\left\{q_{Sa}^{t},p_{Sa}^{t},d_{Sa}^{t}\right\}$, *Ta* calculates out a counter-offer $o_{Ta}^{t}=\left\{q_{Ta}^{t},p_{Ta}^{t},d_{Ta}^{t}\right\}$ with the outcome of team game, where $q_{Ta}^{t}=q_{Sa}^{t}$, $p_{Ta}^{t}$ is calculated by (3) and $D_{Ta}^{t}=\underset{i\in \left\{Pa,La\right\}}{\sum }d_{i}^{t},\;d_{i}^{t}\;\left(i\in \left\{Pa,La\right\}\right)$ is the delivery time given by *Pa* or *La* from their point of view.

**Figure 2:**
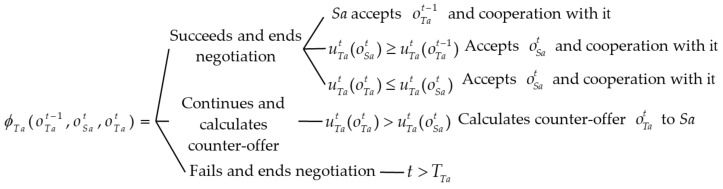
The negotiation strategy of *Ta*. $o_{Ta}^{t-1}=\left\{q_{Ta}^{t-1},p_{Ta}^{t-1},d_{Ta}^{t-1}\right\}$ is the counter-offer of *Ta* at the (*t* − 1)-th round.

To facilitate the negotiation, a complete *negotiation strategy* is defined as a set of rules in a decision tree and designated by *Ta* before starting the automated negotiation. Figure 2 demonstrates a typical negotiation strategy of *Ta*.

#### 2.2.2. Members of Allied Team

During the negotiation, the benefit equilibrium among team members is of delivery time, each member agent selects a suitable delivery time by itself for the *Sa* offer ($o_{Sa}^{t}=\left\{q_{Sa}^{t},p_{Sa}^{t},d_{Sa}^{t}\right\}$) at the *t-*th round, and then calculates the corresponding cost. Provided that the quantity requested by *Sa* will not be altered while member agents calculating their counter-offers, i.e., $q_{Ta}^{t}=q_{SA}^{t}$; a member agent i∈{Pa,La) in *Ta* can be defined as follows:

(7) $$i=\left({\bar{q}}_{i},{\underline{q}}_{i},{\bar{d}}_{i},{\underline{d}}_{i},c_{i},u_{i}\right),\forall i\in \left\{Pa,La\right\}$$

where *u_i_* is the profit evaluation function of *i*:

(8) $$u_{i}^{t}=q_{Sa}^{t}\left(\alpha_{i}p_{Ta}^{t}-C{\;}_{i}^{t}+\beta_{i}\rho_{Ta}^{t}\right),\forall i\in \left\{Pa,La\right\},t=1,\ldots ,T_{Ta}$$

where *α_i_* is the distribution rate of unit price for agent *i*, and set $\alpha_{i}=\frac{c_{i}}{c_{1}+c_{2}}$; *β_i_* is the distribution rate of team collaboration profit for agent *i*, it is calculated based on the Shapley value solution; $p_{Ta}^{t}$is the unit price of *Ta* at the *t-*th round; $\rho_{Ta}^{t}$ is the collaboration profit over the *Ta* counter-offer at the *t-*th round; $C{\;}_{i}^{t}$ is the unit cost of agent *i* for its offer at the *t-*th round [4]:

(9) $$C{\;}_{i}^{t}(d{\;}_{i}^{t},p_{Ta}^{t},q_{Sa}^{t})=\left(1-\delta {\;}_{i}^{t}\right)\left(1+\varepsilon_{i}^{t}\right)c_{i},\forall i\in \left\{Pa,La\right\},t=1,\ldots ,T_{Ta}$$

where *c_i_* is the basic unit cost of agent *i* in general situation, and $c_{i}<\alpha_{i}p_{Ta}^{t};$ $\delta_{i}^{t}\left(0\leq \delta_{i}^{t}<1\right)$ is the influence factor of quantity on the unit cost of agent *i*, and $\delta_{i}^{t}=0$ if $0<q_{Sa}^{t}\leq {\underline{q}}_{i}$, $\delta_{i}^{t}=\frac{q_{Sa}^{t}-{\underline{q}}_{i}}{{\bar{q}}_{i}-{\underline{q}}_{i}}$ if ${\underline{q}}_{t}<q_{Sa}^{t}\leq {\bar{q}}_{i}$; $\varepsilon_{i}^{t}$ is the influence factor of delivery time on the unit cost of agent *i*, and $\varepsilon_{i}^{t}={\left(\frac{{\bar{d}}_{i}-d_{i}^{t}-e_{k}d_{k}^{t}-\sigma_{i}^{t}}{{\bar{d}}_{i}-{\underline{d}}_{i}}\right)}^{2},\;{\underline{d}}_{i}\leq d_{i}^{t}<{\bar{d}}_{i},i,k\in \left\{Pa,La\right\},i\in \neq k$ where $e_{k}=\;\frac{1}{{\bar{d}}_{i}-{\underline{d}}_{i}}$ denotes the correlation factor of the delivery time of agent *k* on agent *i*, and $\sigma_{i}^{t}$ represents the correlation factor of historical negotiation information, and $\sigma_{i}^{t}=0$ if *t* ≤ 2, $\sigma_{i}^{t}=d_{t}^{t-1}\left(\beta_{i}^{t-1}-\beta_{i}^{t-2}\right)$ if 2 < *t* ≤ *T_Ta_*.

### 2.3. Definition of Sa

For the numerical simulation in Section 4, the agent *Sa* of the Stakeholder enterprise is defined as follows:

(10) $$Sa=(W_{Sa},{\bar{o}}_{Sa},{\underline{o}}_{Sa},T_{Sa},\theta_{Sa},\delta_{Sa},v_{Sa},\phi_{Sa},ED_{Sa})$$

where $W_{Sa}=\left\{\omega_{Sa}^{q},\omega_{Sa}^{d},\omega_{Sa}^{p}\right\}$ is the weights set of *Sa* for each issue, and $\omega_{Sa}^{q}+\omega_{Sa}^{d}+\omega_{Sa}^{p}=1$; ${\bar{o}}_{Sa}=\left\{{\bar{q}}_{Sa},{\bar{d}}_{Sa},{\bar{p}}_{Sa}\right\}$ and ${\underline{o}}_{Sa}=\left\{{\underline{q}}_{Sa},{\underline{d}}_{Sa},{\underline{p}}_{Sa}\right\}$ are the sets of ideal and values for each issue; *T_Sa_* is the negotiation deadline acceptable by *Sa*; *θ_Sa_* is the threshold value of satisfaction level, i.e., the minimum of satisfaction level acceptable by *Sa*; *δ_Sa_* is the concession coefficient of *Sa* in calculating offer, and *Sa* chooses it basing on the time and quantity in negotiation [40,41], so $q_{Sa}^{t}={\bar{q}}_{Sa}+\left({\underline{q}}_{Sa}-{\bar{q}}_{Sa}\right){\left(\frac{t-1}{T_{Sa}}\right)}^{\frac{1}{\delta_{Sa}}}$; *v_Sa_* is the offer evaluation function. Therefore, the satisfaction level of $o_{Ta}^{t}$can be calculated with Multiple Attribute Utility Theory (MAUT):

(11) $$v_{Sa}^{}(o_{Ta}^{t})=\omega_{Sa}^{p}\times \frac{p_{Ta}^{t}-{\bar{p}}_{Sa}}{{\underline{p}}_{Sa}-{\bar{p}}_{Sa}}+\omega_{Sa}^{q}\times \frac{q_{Ta}^{t}-{\bar{q}}_{Sa}}{{\underline{q}}_{Sa}-{\bar{q}}_{Sa}}+\omega_{Sa}^{d}\times \frac{d_{Ta}^{t}-{\bar{d}}_{Sa}}{{\underline{d}}_{Sa}-{\bar{d}}_{Sa}},t\leq T_{Sa}$$

**Figure 3:**
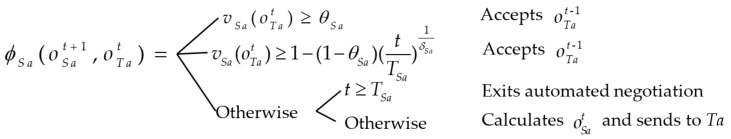
The negotiation strategy of *Sa.*

*φ_Sa_* is the negotiation strategy of *Sa,* as shown in Figure 3:

*ED_Sa_* in (10) is the optimization model [28] of *Sa* to determine its offer by minimizing the Euclidean Distance:

(12) $$\mathrm{Min}\;Ed\left[\left(d_{Sa}^{t},p_{Sa}^{t}\right),\left(d_{Ta}^{t-1},p_{Ta}^{t-1}\right)\right]$$

(13) $$\mathrm{s.t.}\;v_{Sa}\left(o_{Sa}^{t}\right)=1-\left(1-\theta_{Sa}\right){\left(\frac{t}{T_{Sa}}\right)}^{\frac{1}{\delta_{Sa}}}$$

(14) $${\bar{p}}_{Sa}\leq p_{Sa}^{t}\leq {\underline{p}}_{Sa}$$

(15) $${\bar{d}}_{Sa}\leq d_{Sa}^{t}\leq {\underline{d}}_{Sa}$$

(16) $$2\leq t\leq T_{Sa}$$

## 3. Collaborative Game Process of Allied Team

To reflect the characteristics of the vertical allied team with co-opetition partnership, in this paper a game process is designed with non-cooperative game and Shapley Value method.

### 3.1. Counter-Offer Calculation of Team Members

Since there are usually profit conflicts between the two team members, they are not completely cooperative in negotiation and may take some strategic actions to protect their own profit. In the study, the process of calculating the optimal delivery times of *Pa and La* ($d_{Pa}^{t*}$ and $d_{La}^{t*}$) is modeled by a non-cooperative game. The feasible set *D_i_* and the profit evaluation function *u_i_* may determine the strategies of i∈{Pa,La}, and the reaction function $r_{i}:\left(D_{k}\right)\to D_{i},i,k\in \left\{Pa,La\right\},i\neq k$ can be used to find the optimal delivery times for *Pa* and *La*. Assuming that *u_i_* is differentiable, strictly convex and bounded, i.e., the best actions of *i* can be derived from the feasible set *D_i_*. Therefore, with respect to $d_{i}^{t}$ let the first order partial derivative of *u_i_* be zero (∀i∈{Pa,La}), giving us:

(17) $$\frac{\partial u_{i}^{t}}{\partial d_{i}^{t}}=q_{Sa}^{t}\times \left(\alpha_{i}\times \frac{\partial p_{Ta}^{t}}{\partial d_{i}^{t}}-\frac{\partial C_{i}^{t}}{\partial d_{i}^{t}}+\beta_{i}\times \frac{\partial \rho^{t}}{\partial d_{i}^{t}}\right)=0,\forall i\in \left\{Pa,La\right\},t=1,\ldots ,T_{Ta}$$

It is not difficult to find that the cross point of the reaction functions is the Nash equilibrium solution for the game between *Pa* and *La* of calculating the counter-offer, i.e., no member can benefit by changing its delivery time while the other members keep theirs unchanged when $q_{Sa}^{t}$ is fixed. By the arrangements of (17), we can get:

(18) $$d_{i}^{t}=\mu_{i}^{t}-\alpha_{i}\frac{\lambda_{}^{t}}{\xi_{i}^{t}}-e_{k}d_{k}^{t},\forall i,k\in \{Pa,La\},i\neq k,t=1,\ldots ,T_{Ta}$$

where $\lambda^{t}=\frac{1}{4}\frac{b_{Ta}\Delta_{Ta}^{t}}{{\left({\bar{d}}_{nt}-{\underline{d}}_{i}\right)}^{2}}$, $\xi_{i}^{t}=\frac{\left(1-\delta_{i}^{t}\right)c_{i}}{{\left({\bar{d}}_{i}-{\underline{d}}_{i}\right)}^{2}}$ and $\mu_{i}^{t}={\bar{d}}_{i}-\sigma_{i}^{t}$.

By solving the above two equations system, the optimal delivery times of *Pa* and *La* at the *t-*th round may be calculated:

(19) $$d_{i}^{t*}=\frac{\mu_{i}^{t}-\alpha_{i}\frac{\lambda_{}^{t}}{\xi_{i}^{t}}-\mu_{k}^{t}e_{k}+\alpha_{k}e_{k}\frac{\lambda_{}^{t}}{\xi_{k}^{t}}}{1-e_{i}e_{k}},\forall i,k\in \{Pa,La\},i\neq k,t=1,\ldots ,T_{Ta}$$

where $\xi_{k}^{t}=\frac{\left(1-\delta_{k}^{t}\right)c_{k}}{{\left({\bar{d}}_{k}-{\underline{d}}_{k}\right)}^{2}}$ and $\mu_{k}^{t}={\bar{d}}_{k}-\sigma_{k}^{t}$.

Then, by substituting (19) into (8), we can get the optimal profit values of *Pa* and *La* in terms of $q_{Sa}^{t}:$ $u_{Pa}^{t*}$ and $u_{Sa}^{t*}$ respectively.

### 3.2. Shapley Value based Distribution Rate of Collaboration Profit

During the negotiation, the collaboration profit of *Ta* will be distributed to *Pa* and *La* according to the contribution rates of their offers to the total profit. In this study, the contribution rates are calculated with Shapley value [42,43]. When all member agents of the team are fully cooperative with each other, the form of a cooperative game can be represented as (*N,v*), where *N =* {1,…,*n*} is the set of member agents, and *v*:2*^N^*→*R* is a profit function of an offer in negotiation. An intuitive interpretation of the Shapley value can be described as follows: considering all possible ranking sequences of member agents, a marginal contribution of member agent *i* is defined with respect to a given ranking sequence as its marginal worth to the team as its existence in the sequence, i.e., *v*({1,…, *i*−1, *i*})−*v*({1,…, *i−*1}), where {1,…, *i−*1} are the team member agents preceding *i* in the given ranking sequence. Thus, the Shapley value is obtained by averaging the marginal contributions for all possible sequences as shown in the following formula [44]:

(20) $$\pi_{i}(v)=\sum_{\left\{S:i\in S\right\}}\frac{(|S|-1)!(n-|S|)!}{n!}(v(S)-v(S\backslash \{i\}))$$

where *v*(*S*) denotes the profit generated by the offer of partial team S∈N. As the team only has two agent (*Pa* and *La*) in the study (i.e., *N* = {1,2}), and {1} and {2} represent *Pa* and *La*, respectively, the Shapley values of *Pa* and *La* offers can be expressed as follows [45,46,47,48]:

(21) $$\pi_{Pa}^{t}(v)=\frac{(1-1)!(2-1)!}{2!}(v(\{1\})-v(\{1\}-\{1\}))+\frac{(2-1)!(2-2)!}{2!}(v(\{1,2\})-v(\{1,2\}-\{1\}))$$

(22) $$\pi_{La}^{t}(v)=\frac{(1-1)!(2-1)!}{2!}(v(\{2\})-v(\{2\}-\{2\}))+\frac{(2-1)!(2-2)!}{2!}(v(\{1,2\})-v(\{1,2\}-\{2\}))$$

where $v(\{1\})={\tilde{u}}_{Pa}^{t*}$, $v(\{2\})={\tilde{u}}_{La}^{t*}$, $v(\{1,2\})=u_{Ta}^{t*}$, and v(0)=0; ${\tilde{u}}_{Pa}^{t*}$ and ${\tilde{u}}_{La}^{t*}$ denote the profits of *Pa* and *La* obtained from their own offers if they did not join in the allied team, and ${\tilde{u}}_{i}^{t*}=u_{i}^{t*}-\beta_{i}\rho_{Ta}^{t},\forall i\in \{Pa,La\},t=1,\ldots ,T_{Ta}$. In contrast, $u_{Ta}^{t*}$ denotes the total profit that *Ta* gets if *Pa* and *La* join in the team, and $u_{Ta}^{t*}=\;u_{Pa}^{t*}+u_{La}^{t*}=\;{\tilde{u}}_{Pa}^{t*}+{\tilde{u}}_{La}^{t*}+\rho_{Ta}^{t}$, $d_{Ta}^{t*}=\;d_{Pa}^{t*}+\;d_{La}^{t*},\forall t=1,\ldots ,T_{Ta}$. Hence, by arranging (21) and (22), we have:

(23) $$\pi_{Pa}^{t}(v)=\frac{1}{2}(v\{1\}+v\{1,2\}-v\{2\})=\frac{1}{2}({\tilde{u}}_{Pa}^{t*}+u_{Ta}^{t*}-{\tilde{u}}_{La}^{t*})$$

(24) $$\pi_{La}^{t}(v)=\frac{1}{2}(v\{2\}+v\{1,2\}-v\{1\})=\frac{1}{2}({\tilde{u}}_{La}^{t*}+u_{Ta}^{t*}-{\tilde{u}}_{Pa}^{t*})$$

Therefore, the distribution rates of collaboration profit with respect to *Pa* and *La* in the team can be expressed as: $\beta {\;}_{Pa}^{t}=\frac{\pi_{Pa}^{t}(v)}{\pi_{Pa}^{t}(v)+\pi_{La}^{t}(v)}=\frac{1}{2}\times \frac{{\tilde{u}}_{Pa}^{t*}+u_{Ta}^{t*}-{\tilde{u}}_{La}^{t*}}{u_{Ta}^{t*}}$ and $\beta {\;}_{La}^{t}=\frac{\pi_{La}^{t}(v)}{\pi_{Pa}^{t}(v)+\pi_{La}^{t}(v)}=\frac{1}{2}\times \frac{{\tilde{u}}_{La}^{t*}+u_{Ta}^{t*}-{\tilde{u}}_{Pa}^{t*}}{u_{Ta}^{t*}}$, which depend on the offers of *Pa* and *La*.

**Figure 4:**
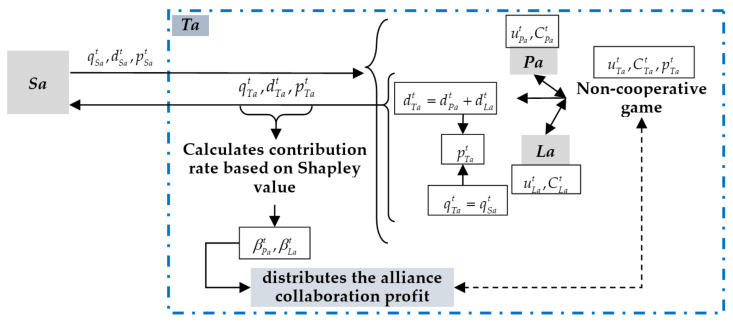
The team game process for calculating counter-offers.

The process of team game between *Pa* and *La* in *Ta* is shown in Figure 4. In the negotiation of each round, *Pa* and *La* calculate their own offers based on the non-cooperative game, and then *Ta* distributes the collaboration profit according to their contribution rates, and the automated negotiation iterates round by round until the termination criteria is met. *Pa* and *La* may take the profit of *Ta* and their individual profits into account of their offers. Notably, in first round, the distribution of collaboration profit is initialized with fixed rates.

## 4. Numerical Simulation and Analysis

### 4.1. Simulation Experiments

In recent years, China’s energy transition to tackle climate change, from a coal-dominated system to one with the world largest deployment of renewable energy such as wind or solar energy, has intensified the dual pressures of the coal enterprises including cutting overcapacity and fierce competition in coal industry as China entered new normal economy [49]. To enhance the operational efficiency, improve the service level for customers and maintain the competition edges in market, many coal production enterprises often group a vertical alliance with coal logistics enterprises or other enterprises in the supply chain as a business tactic in their competitive strategy [50]. In this study, two members of alliance with one *Production enterprise* and one *Logistics enterprise*, and one *Stakeholder enterprise* are involved in the numerical simulation. With the notations designated in previous sections, the *Production enterprise* entity is a large coal production enterprise in Inner Mongolia, which seeks to outsource the coal logistics, to enhance the production capacity, efficiency and quality. The *Logistics enterprise* entity is a professional logistics enterprise which engages in coal transportation. The *Stakeholder enterprise* entity is a large power & heating company in Guangzhou, which sooner or later replenishes coal for power generation, a power & heating company needs to replenish a quantity of coal of specifications at some costs (the unit calorific value should be between 6300 and 6400 Kcal, moreover, the volatile matter, total sulfur and moisture should be less than 14%, 0.9% and 8% respectively. The known conditions include that the distance is about 25,000 Km, and logistics cost has a considerable proportion in the cost of sales (at least 60% in the sale cost of coal according to the objective statistics [39,50])), and sends the request to the B2B electronic platform. To support the power & heating company, the B2B electronic platform groups a coal production enterprise and a professional logistics enterprise as an allied negotiation team in some ways (*the supply chain B2B electronic platform* may find the team with some rules or strategies, in a process that might need qualified *Production enterprise* and *Logistics enterprise* to interact with each other and confirm the team formation), to negotiate with the power & heating company for making a supply contract or agreement. The B2B electronic platform initializes four agents: *Sa*, *Pa*, *La*, *Ta*, accordingly for the power & heating company, the coal production enterprise, the *professional* logistics enterprise and the allied team, and then starts an automated negotiation process designed as above, and the issues of negotiation involve quantity *q* (ton), unit price *p* (RMB/ton) and delivery time *d* (due day), i.e., the issues set of negotiation is {*q*, *d*, *p*}. Assuming that, all member agents of the team independently make their decision and at the same time, there is no leader or follower in the team about making offers during the negotiation with *Sa*. Each of *Pa* and *La* is economically rational and acts strategically, usually seeks for maximizing its own profits. All team member agents have the common cost function but with different parameters setting, for more profits they may share some information or resources and the team settings are in common sense such as the pricing rules, collaboration profit function and the historical information of the negotiation.

### 4.2. Parameter Settings

To testify the effectiveness of the proposed model, all parameters of *Ta, Pa*, *La* and *Sa* are set randomly in rational ranges as Table 2, Table 3 and Table 4.

### 4.3. Analysis of Experimental results

Based on the settings in Table 2, Table 3 and Table 4, a set of illustrative parametric data of a problem instance is shown in Table 5.

During the automated negotiation, to maximize their individual profits, *Pa* and *La* may calculate their best offers according to their own situation. With regards to the offer of *Sa*, the counter-offer of *Ta* is not mandatorily assigned, but each member agent (*Pa* and *La*) participates in the decision-making, which is constructive for the operational fulfilment in future. Moreover, the distribution of collaboration profit based on Shapley value has leaded to each member agent doing best to make the total profit of *Ta*.

**Figure 5:**
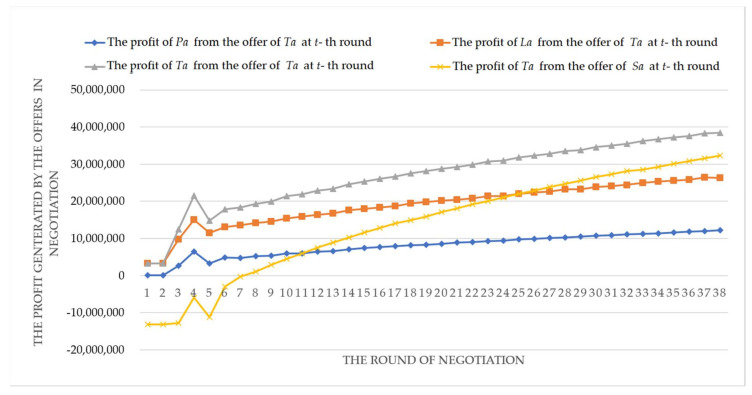
The profits of *Ta* and its member agents during the negotiation.

**Figure 6:**
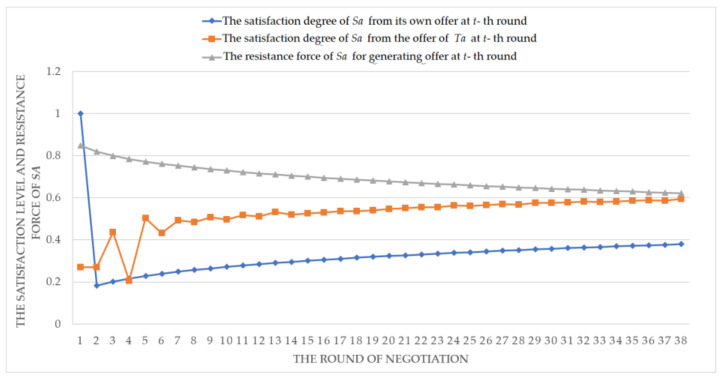
The satisfaction level and resistance force of *Sa* during the negotiation.

In Figure 5, it is shown that the profits of *Ta*, *Pa* and *La* are consistently improved in the negotiation process. Meanwhile, Figure 6 shows that the profit improvement of *Ta* is not relying on the loss of *Sa*, but improves the satisfaction level of *Sa* accordingly. Finally, at the 38th round, by the negotiation strategy in Figure 4, *Sa* accepts the offer of *Ta*: $o_{Ta}^{38}=\left\{45160.56,\;55,\;601.51\right\}$, (i.e., quantity is 45,160.56 ton, delivery time is 55 days, and unit price is 601.51 Yuan/ton), because the satisfaction level of *Sa* is 0.5943 which is bigger than its threshold *θ_Sa_* (0.59). The profits of *Ta*, *Pa* and *La* are 38,467,922.45 Yuan, 12,168,691.6 Yuan and 26,299,230.84 Yuan, respectively. As a result, we can find that *ANT* is suitable to solve the trading negotiation problems between allied vertical alliance and stakeholder enterprise, where a win-win effective agreement or contract can be obtained.

**Figure 7:**
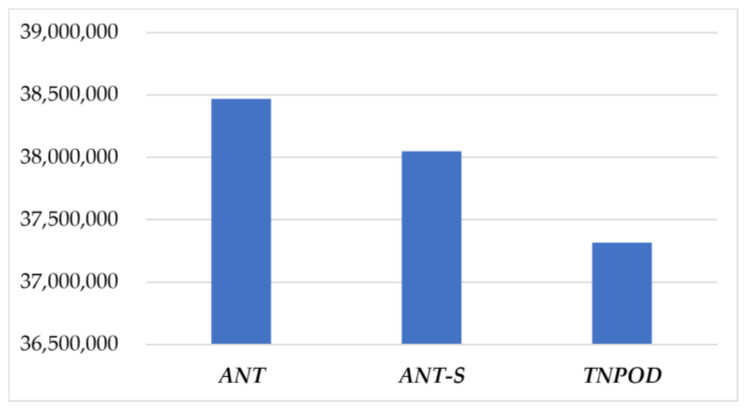
The comparison of *Ta*’s profit by different models.

To compare *ANT* with other models of different mechanisms: *ANT-S* (The distribution of collaboration profit in *ANT* based on a fixed rate) and *TNPOD* (Sanchez-Anguix et al. [26,28]), with data in Table 5. The comparison of the models is shown in Figure 7.

Through the simulation experiments, it is found that *ANT-S* and *TNPOD* can also succeed in the negotiation within 38 rounds, but the obtained profits of *Ta* are significantly different, as shown in Figure 7. By *ANT-S*, the maximum profit of *Ta* (38,049,564.59 Yuan) is 418,357.86 Yuan less than *ANT* (38,467,922.45 Yuan), indicating that the profit distribution by the Shapley value in *ANT* is more effective than those on a fixed distribution rate. By *TNPOD*, the final profit of *Ta* is 37,317,253.12 Yuan, less 1,150,669.33 and 732,311.47 than *ANT* and *ANT-S* respectively, implying that the team game-based method may obtain higher profit for *Ta* than the voting-based method.

**Figure 8:**
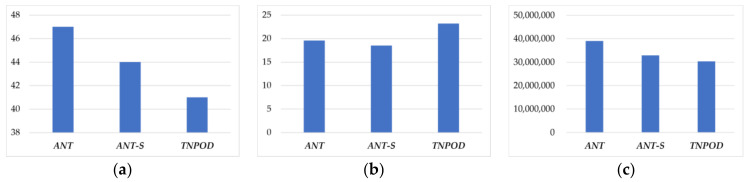
The average results comparison of 50 times simulations. (**a**) Number of successful negotiations; (**b**) Number of negotiation rounds; (**c**) Average profit.

Moreover, to further verify the effectiveness of *ANT*, this study has compared the average results of 50 times simulations of *ANT* with *ANT-S* and *TNPOD*. The comparison results are shown in Figure 8.

In Figure 8a, it is shown that *ANT* can obtain more successful negotiations than *ANT-S* and *TNPOD* in the 50 simulation experiments, which indicates that *ANT* has better adaptability to negotiation environment and instance parameters. In Figure 8b, the average number of negotiation rounds is slightly worse than *ANT-S*, but is better than *TNPOD*. The main reason for this is that *ANT* may make member agents more prudent in calculating offers than *ANT-S* to obtain more collaboration profit, and *TNPOD* allows *Ta* more offer adjustments for a mutually acceptable agreement in negotiation. Fortunately, the gap of rounds between *ANT* and *ANT-S* is not significant, so it has not obvious effect on the result of the undergoing automatic negotiation processes. In Figure 8c, the average profit of *Ta* is obviously better than *ANT-S* and *TNPOD*, like the result of simulation experiment in Figure 7. Notably that, in Figure 8 the average number of the negotiation rounds, and the average profit of *Ta* are obtained in successful negotiations.

In summary, in terms of the above numerical simulation results and analysis, the proposed *ANT* can be effective and helpful for the allied team and stakeholder to make a business contract or agreement, through the automated negotiation on a supply chain B2B electronic platform.

## 5. Conclusions

With the emergence of the new normal economy, the market competition is increasingly fierce. To keep their competitive edge, enterprises often group vertical alliance with upstream and downstream partners in supply chain as a business tactic in their competitive strategy. Although, in reality, allied members do expect that their profits can be improved by the cooperation in an alliance, it is difficult for them to completely share resources and operational information due to privacy or trust consideration. To improve their bargaining competence in the negotiation with opponents, in terms of cost reduction of operations, trading and technology innovation, the members of the vertical alliance may form as a single negotiation team. Considering the characteristics that the competition and cooperation coexist in the team, this paper designed automated negotiation model and a team game process, aiming at obtaining a business agreement on the fulfilment of stakeholder’s demand. In *ANT*, the offer calculation in the allied team is described and analyzed from the perspective of non-cooperative game, and solved with Shapley value, so that the collaboration profit is distributed among team members according to their contribution rates. Through a case study of simulation experiments with a power & heating company and an allied team with a coal enterprise and a logistics enterprise, the effectiveness of *ANT* has been testified. 

The research shed light on the cooperation among the vertical supply chain and demand stakeholder: (1) although the vertical allied team in a negotiation may significantly improve their bargaining power, the decision-making mechanism of the counter-offer may influence the final profit of the team; (2) the team formation and the decision-making mechanism need take into account the characteristics of the vertical supply chain with co-opetition; (3) the decision-making mechanism embedded with suitable game process may improve the efficiency and quality of the counter-offer attainable by the team; (4) the combination of cooperative and non-cooperative games may be a good way to describe the decision-making processes in the concerned problem.

Our research makes some contributions on theoretic and practical applications: (1) the decision-making of the vertical allied team with co-opetitive relationship is shaped with game process in the negotiation with opponent; (2) with the proposed automated negotiation model, the vertical allied team may properly manage internal collaborative business even member competition coexists; (3) this model can help allied teams, in a supply chain or other independent organization that cooperate for fulfilling a task, to make an agreement with other stakeholders or potential cooperators, with the assumption that team members may have different know-how or roles for fulfilling the tasks but not affect the decision-making in the team, and the tasks are not so clearly divided among the team members, meanwhile the working approaches are less rigid and eventually more prone to lower the work efficiency or generate much more extra cost as the inferior collaboration. By appropriate improvement or adjustment, the proposed model can be applied to solving problems in other domains that may be supported by agent-based negotiation teams, such as the *team-based services finding*, for instance, in the travel service platform such as *TripAdvisor* (an American travel and restaurant platform company providing hotel and restaurant reviews, accommodation bookings and other travel-related content.), and the *team-based management of complex democratic organization*, for instance, the agricultural cooperatives reported in the literature [51] such as the agricultural products platform such as *Hello Fresh* and *Blue Apron* in American and so on.

From the two practical application scenarios, it is observable that an electronic marketplace platform is necessary for a physical connection between members to make information sharing feasible for various flow activities. Although the work flow of the proposed model aims to be general and adaptable to a wide variety of domains and applications, some improvement or adjustment must be done to meet the specific demands of different platform environments. Moreover, In the present research many practical factors such as the enterprise scale and technological superiority, etc. don’t affect the decision-making of member agents in the vertical allied enterprises team, which may be impractical. Thus, subsequent research should be based on this foundation and further studies the influence of the specific team with distinct size or structure, in which member enterprises might have different know-how and therefore some of them might be naturally in a more advantageous position than other companies. In future research, the model might also be improved by considering more negotiation issues besides the delivery time and other negotiation situations such as three or more teams involved in a negotiation. In addition, some unique challenges like how to form a negotiation team and how to shape and define the cost or profit function of agents should be considered with the new topic.

## Figures and Tables

**Table 1:** The Notations and Descriptions in *ANT.*

Notations	Descriptions
*Pa*, *La*	the agents delegating *Production enterprise* and *Logistics enterprise* in an automated negotiation
${\bar{q}}_{Ta}$, ${\underline{q}}_{Ta}$	the ideal and threshold value for quantity (${\bar{q}}_{Ta}>{\underline{q}}_{Ta}$)
${\bar{d}}_{Ta}$, ${\underline{d}}_{Ta}$	the ideal and threshold value for delivery time (${\bar{d}}_{Ta}>{\underline{d}}_{Ta}$)
$p_{Ta}$	the round-dependent price function
$b_{Ta}$	the average unit price in the market, and we set *b_Ta_* = (1 + *γ_b_*)(*c_Pa_* + *c_La_*) in this paper
$\gamma_{b}$	the general markup percentage of price relative to total cost value
$T_{Ta}$	the deadline designated when the automated negotiation is initialized
$\Delta_{Ta}^{t}$	the influence factor of the quantity on unit price, $0\leq \Delta_{Ta}^{t}\leq 1$
$\eta_{Ta}^{t}$	the influence factor of the delivery times of *Pa* and *La* on unit price
$D_{Ta}^{t}$	the delivery time requested by *Ta* at the *t*th round, $D_{Ta}^{t}=\underset{i\in \left\{Pa,\;\;La\right\}}{\sum }d_{i}^{t},\forall t=1,\ldots ,T_{Ta}$
$\rho_{Ta}^{t}$	*the alliance collaboration profit* from the team offer at the *t*-th round
$\alpha_{Ta}$	the distribution rates set of $p_{Ta}^{t}$ for *Pa* and *La*, *α_Ta_* = {*α_Pa_*,*α_La_*} and *α_Pa_* + *α_La_* = 1
$\beta_{Ta}$	the distribution rates set of $\rho_{Ta}^{t}$ for *Pa* and *La*, *β_Ta_* = {*β_Pa_*,*β_La_*} and *β_Pa_* + *β_La_* = 1
$\phi_{Ta}$	the negotiation strategy of *Ta*
${\bar{q}}_{i}$, ${\underline{q}}_{i}$	the ideal and threshold quantities of agent *i*, ${\bar{q}}_{i}>{\underline{q}}_{i}$, ∀i={Pa,La}
${\bar{d}}_{i}$, ${\underline{d}}_{i}$	the ideal and threshold deliver times of agent *i*, ${\bar{d}}_{i}>{\underline{d}}_{i}$, ∀i={Pa,La}
$d{\;}_{i}^{t}$	the delivery time requested by agent *i*, ∀i={Pa,La}
$C{\;}_{i}^{t}$	the unit cost of agent *i* with the team offer at the *t*th round, ∀i={Pa,La}
$c_{i}$	the basic unit cost of agent *i* in general case, ∀i={Pa,La}
$\delta_{i}^{t}$	the influence factor of quantity on the unit cost of agent *i*, $0\leq \delta_{i}^{t}<1$, ∀i={Pa,La}
$\varepsilon_{i}^{t}$	the influence factor of delivery time on the unit cost of agent *i*, ∀i={Pa,La}
$\sigma_{i}^{t}$	the correlation factor of historical negotiation information for agent *i*, ∀i={Pa,La}

**Table 2:** The data template for simulating *Ta.*

Parameters	Value Ranges	Parameters	Value Ranges
${\bar{q}}_{Ta}$	[30000,50000]	${\underline{q}}_{Ta}$	${\bar{q}}_{Ta}-15000$
${\bar{d}}_{Ta}$	[125,110]	${\underline{d}}_{Ta}$	${\bar{d}}_{Ta}-40$
${\bar{p}}_{Ta}$	${\underline{p}}_{Ta}+500$	${\underline{p}}_{Ta}$	[550,650]
$T_{Ta}$	[20,50]	$\gamma_{b}$	[0.2,0.35]

**Table 3:** The data template for simulating *Pa* and *La.*

Parameters	Value Ranges	Parameters	Value Ranges
${\bar{q}}_{Pa}$	${\bar{q}}_{Ta}+5000$	${\bar{q}}_{La}$	${\bar{q}}_{Ta}$
${\underline{q}}_{Pa}$	${\underline{q}}_{Ta}$	${\underline{q}}_{La}$	${\underline{q}}_{Ta}-3000$
${\bar{d}}_{Pa}$	[60,75]	${\bar{d}}_{La}$	[50,65]
${\underline{d}}_{Pa}$	${\bar{d}}_{Pa}-25$	${\underline{d}}_{La}$	${\bar{d}}_{La}-30$
$c_{Pa}$	[500,550]	$c_{La}$	[600,650]

**Table 4:** The data template for simulating *Sa.*

Parameters	Value Ranges	Parameters	Value Ranges
${\bar{p}}_{Sa}$	[550,650]	${\underline{p}}_{Sa}$	${\bar{p}}_{Sa}+500$
${\bar{q}}_{Sa}$	[15000,35000]	${\underline{q}}_{Sa}$	${\bar{q}}_{Sa}+15,000$
${\bar{d}}_{Sa}$	[35,40]	${\underline{d}}_{Sa}$	${\bar{d}}_{Sa}+30$
$\theta_{Sa}$	[0.45,0.65]	$\delta_{Sa}$	[2,15]
$T_{Sa}$	$T_{Ta}+5$		

**Table 5:** Illustrative parametric data of problem instance.

Parameters	Value	Parameters	Value	Parameters	Value	Parameters	Value
${\bar{q}}_{Ta}$	46,384	${\underline{q}}_{Ta}$	31,384	${\bar{d}}_{Ta}$	119	${\underline{d}}_{Ta}$	79
${\bar{q}}_{Pa}$	51,384	${\underline{q}}_{Pa}$	31,384	${\bar{d}}_{Pa}$	67	${\underline{d}}_{Pa}$	42
${\bar{q}}_{La}$	46,384	${\underline{q}}_{La}$	28,384	${\bar{d}}_{La}$	57	${\underline{d}}_{La}$	37
$T_{Ta}$	47	$\gamma_{b}$	0.32	$c_{Pa}$	514	$c_{La}$	623
${\bar{q}}_{Sa}$	31,384	${\underline{q}}_{Sa}$	46,384	${\bar{d}}_{Sa}$	39	${\underline{d}}_{Sa}$	69
${\bar{p}}_{Sa}$	578	${\underline{p}}_{Sa}$	1078	$\theta_{Sa}$	0.59	$\delta_{Sa}$	4
$T_{Sa}$	52	$\omega_{Sa}^{q}$	0.30	$\omega_{Sa}^{d}$	0.20	$\omega_{Sa}^{p}$	0.50
